# Dynamics of the Honeybee (*Apis mellifera*) Gut Microbiota Throughout the Overwintering Period in Canada

**DOI:** 10.3390/microorganisms8081146

**Published:** 2020-07-29

**Authors:** Naomie Bleau, Sidki Bouslama, Pierre Giovenazzo, Nicolas Derome

**Affiliations:** 1 Biology Departement, Laval University, 1045 Avenue de la Médecine, Quebec City, QC G1V 0A6, Canada; sidki.bouslama.1@ulaval.ca (S.B.); pierre.giovenazzo@bio.ulaval.ca (P.G.); nicolas.derome@bio.ulaval.ca (N.D.); 2 Centre de Recherche en Sciences Animales de Deschambault (CRSAD), 120a Chemin du Roy, Deschambault, QC G0A 1S0, Canada; 3 Institut de Biologie Intégrative et des Systèmes (IBIS), Laval University, 1030 Avenue de la Médecine, Quebec City, QC G1V 0A6, Canada

**Keywords:** microbiome, gut dysbiosis, symbiont, winter, pollinator

## Abstract

Microbial symbionts inhabiting the honeybee gut (i.e., gut microbiota) are essential for food digestion, immunity, and gut protection of their host. The taxonomic composition of the gut microbiota is dynamic throughout the honeybee life cycle and the foraging season. However, it remains unclear how drastic changes occurring in winter, such as food shortage and cold weather, impact gut microbiota dynamics. The objective of this study was to characterize the gut microbiota of the honeybee during the overwintering period in a northern temperate climate in Canada. The microbiota of nine honeybee colonies was characterized by metataxonomy of 16S rDNA between September 2017 and June 2018. Overall, the results showed that microbiota taxonomic composition experienced major compositional shifts in fall and spring. From September to November, *Enterobacteriaceae* decreased, while *Neisseriaceae* increased. From April to June, *Orbaceae* increased, whereas *Rhizobiaceae* nearly disappeared. Bacterial diversity of the gut microbiota decreased drastically before and after overwintering, but it remained stable during winter. We conclude that the honeybee gut microbiota is likely to be impacted by the important meteorological and dietary changes that take place before and after the overwintering period. Laboratory trials are needed to determine how the observed variations affect the honeybee health.

## 1. Introduction

All animals, including insects, host a variety of microorganisms (bacteria, archaea, yeast, viruses) in their digestive tract that is defined as their gut microbiota [1]. The honeybee (*Apis mellifera*) harbors a simple core microbiota in the gut, mostly dominated by six bacterial families: *Lactobacillaceae*, *Acetobacteriaceae* (*Parasaccharibacter apium*), *Rhizobiaceae* (*Bartonella apis*), *Bifidobacteriaceae*, *Neisseriaceae* (*Snodgrassella alvi*) and *Orbaceae* (*Gilliamella apicola*, *Frischella perrara*) [2,3,4].

The relationship between the gut microbiota and its host is mutualistic [5]. The gut offers a safe and nutrient-rich environment for the microbes to thrive, while microorganisms perform many tasks related to nutrition [6,7], immunity [8,9,10] and gut epithelium protection [11]. The honeybee gut is first colonized with microorganisms through contact with nurse bees and various foods during their larval stage. However, during their metamorphosis into pupae, the gut epithelium is excreted with gut bacteria by defecation [12]. The emerging bee is axenic and acquires its mature gut microbiota during its first week of life mostly through trophallaxis, faecal-oral interaction [13] and contact with hive material [14].

During the adult life of the honeybee, factors such as food quality, parasite presence and chemicals impact its microbiota composition and diversity [15]. Asian honeybees (*Apis ceranae*) fed with beebread have a different and more diverse microbiota compared to those fed with plain sugar syrup [16]. Moreover, feeding bees aged pollen causes a reduction of *Snodgrassella alvi* in the honeybee gut, and an increase of *Frischella perrara*, a bacterium associated with impaired development [17]. The access to fresh and diverse food seems essential to maintain a normal and diverse gut microbiota.

In colonies infected by the parasitic mite *Varroa destructor*, there are an increased number of *S. alvi* and a decrease of *Lactobacillaceae* in the gut of workers [18,19]. Additionally, the microbiota of the parasitized larvae is similar to that of *V. destructor* [20]. Artificial infection of bees with *Nosema ceranae*, a pathogenic microsporidium, also alters the microbiota and provokes a rise in the abundance of *Gilliamella apicola* [21]. To control these parasites, beekeepers frequently use chemicals such as fumagilin (against *Nosema* spp.) and oxalic acid (against *V. destructor*). However, these products are known to reduce the diversity and bacterial abundance of the honeybee gut microbiota [22,23].

To explore the combined impact of these factors on the honeybee gut microbiota, Ludvigsen et al. studied the bacterial community of the honeybee midgut during part of a beekeeping season, from May to October. They found that *G. apicola* dominates the midgut in May and is replaced by *S. alvi* later in summer. They also observed a significant increase in diversity between May and June. Their results suggest that the honeybee gut microbiota is mainly driven by food availability and bee demography during the foraging season [14].

However, to our knowledge, the evolution of the honeybee gut microbiota from October to May has not yet been described. In the northern climates typical of north-eastern Canada, overwintering colonies experience unique environmental conditions and must be prepared accordingly. Starting in early fall, colonies are fed concentrated sucrose syrup (2:1) to ensure that they have sufficient food stores. Colonies are then treated against *V. destructor* with various acaricides and *Nosema* spp. with a fungicide, Fumidyl-B^®^. Afterwards, when the average daytime temperature is around 0 °C, colonies are overwintered indoors in a common environmentally controlled room (3–5 °C and 30–40% RH) or outdoors with added insulation. Overwintering usually lasts from early December to April and is a critical period for the Canadian beekeeping industry. In the past 10 years, Canadian beekeepers have experienced large losses, with an average of 26% of their colonies dying while wintering or early in spring [24].

Colony behavior and honeybee physiology change significantly in winter [25]. Overwintering bees have a lifespan of over 100 days, compared to an average of 30 days for summer bees [26]. In winter, they form a compact cluster around their queen and generate heat through flight muscle vibration [27]. Throughout the overwintering period, honeybees are confined within the hive and do not defecate [28]. As a result, *Nosema* spp. spore load increases in the midgut of winter bees, impacting their gut microbiota [21] and health [29].

This combination of environmental, nutritional, and behavioral changes unique to a northern climate could impact the bacterial communities living in the honeybee gut. In this research, we investigated the compositional and structural shifts taking place in worker bees’ gut from September to June in colonies living in north-eastern Canada. We hypothesize that gut microbiota composition and diversity will follow a seasonal trend. We expect that microbial diversity will decrease in fall and winter because of antiparasitic treatments, isolation and the absence of plants to forage, which are an important source of microorganisms [30]. Knowing how the microbial communities are affected by seasonal changes will allow us to identify gut dysbiosis in honeybees and propose probiotic nutritional supplements to improve the health of wintering colonies.

## 2. Materials and Methods

### 2.1. Environment and Colony Management

No permits were required for this research. The study took place at the Centre de Recherche en Sciences Animales de Deschambault and was conducted on nine honeybee colonies of similar strength (total brood area) selected from among the livestock at our bee research facility (CRSAD, Deschambault, QC, Canada; 46°40′30.0″ N, 71°54′52.3″ O). During the setup phase, young sister queens were introduced in the colonies in July 2017. Following acceptance of the queens, the colonies were moved to two apiaries located in Deschambault, Quebec, near the bee research facility. The experimental phase of the project took place between September 2017 and June 2018. At the beginning of September, honey supers were removed, and colonies were reduced to one brood chamber. Fall feeding started in mid-September and all colonies were given 24 L of a sucrose 2:1 solution using a top box feeder (Wooden Miller feeder # FE-1100 from Propolis-etc., Beloeil, QC, Canada). Colonies received a Thymovar anti-varroa treatment starting on September 12, followed by an oxalic acid treatment on November 5 (drip method: 35 g/L in a sucrose 1:1 solution, 5 mL between every frame of the hive body crowded with honeybees). Colonies were wintered indoors in an environmentally controlled room (4–5 °C, 50–60% RH) from 22 November 2017 to 20 April 2018, and then moved to two spring apiaries until the end of June 2018.

### 2.2. Honeybee Sampling

Bees were sampled at four key times: September 6, after the last honey flow; November 2, after the anti-varroa treatments and before entering the wintering room; April 27, after wintering and removal from the wintering room; and June 1, during the first honey flow of the year. Samples consisted of approximately 100 nurse bees shaken off brood frames and immediately freeze-killed on dry ice. Samples were then brought to the lab and stored in a freezer at −86 °C (Thermofisher −86 °C FORMA 908, Waltham, MA, USA).

### 2.3. Sample Preparation and Sequencing

To assess bacterial composition of the honeybees’ microbiota, V3–V4 hyper-variable regions of the 16S small subunit (SSU) rDNA gene were targeted. To this end, the midgut of 20 bees per colony was removed from their abdomen with sterile forceps and pooled together in 2 mL microtubes. Then, 800 µL of sterile salt homogenizing buffer (5M NaCl, 1M Tris-HCl pH 8.0 and 0.5M EDTA) and sterile 4.5 mm metal beads were added to the microtubes. The pooled midguts were vortexed for one minute to homogenize them.

Total DNA was extracted using a salt-extraction method [31]. First, 440 µL of the midgut homogenate and 20 µL of lysozyme (25 mg/L) were mixed and incubated at 37 °C for 1h. Then, 44 µL of 20% SDS and 8 µL of proteinase K (20 mg/mL) were added and vortexed. The samples were incubated at 56 °C overnight, rotating at a speed of 600 rpm. The next day, 4 µL RNase (10 mg/mL) was added to the tubes and incubated for an hour at 37 °C. Then, 300 µL of 6M NaCl was added to each sample before being vortexed (1 min) and centrifuged (20 min, 16,000× *g*, 4 °C). The supernatant was transferred to a new 1.5 mL microtube, and the same volume of cold isopropanol (−20 °C) was added. The tubes were gently mixed and incubated at −20°C. After 30 min, the tubes were centrifuged (20 min, 16,000× *g*, 4 °C) and the supernatant was discarded. Then, 200 µL of cold ethanol (−20 °C) was added to the tubes, followed by a last centrifugation (10 min, 16,000× *g*, 4 °C). The supernatant was discarded once more, and the DNA pellet was left to dry before being placed in 100 µL of DNA- and RNA-free water. The DNA was kept at −20 °C until needed.

The PCR amplification of the V3–V4 region of the bacterial 16S rDNA gene was carried out with the 803R 5′-GTG ACT GGA GTT CAG ACG TGT GCT CTT CCG ATC TCT ACC RGG GTA TCT AAT CC-3′) and 347F (5′-ACA CTC TTT CCC TAC ACG ACG CTC TTC CGA TCT GGA GGC AGC AGT RRG GAA T-3′) primers. The PCR reactions were performed in a total volume of 50 µL containing 10.5 µL sterile water, 3 µL DNA template, 2.5 µL of each primer, 10 µL of Reaction Buffer 5×, 10 µL of GC enhancer 5× and 0.5 µL of Q5 Taq Polymerase (New England Biolabs, Ipswich, MA, USA). The PCR conditions were as follows: denaturation for 2 min at 98 °C, followed by 35 cycles of 10 s at 98 °C, 30 s at 60 °C and 30 s at 72 °C for the amplification, and final extension at 72 °C for 2 min. PCR products (approximately 500 pb) were visualized by electrophoresis in 2% (*w*/*v*) agarose gels and purified with AMPure XP beads (Beckman Coulter Life Sciences, Brea, CA, USA). The quality of amplicons was assessed using a spectrophotometer (NanoDrop2000, ThermoFisher Scientific). A second PCR amplification was conducted to add unique barcodes to every sample. The same steps performed for the first PCR were followed to visualize, purify and quantify the amplicons. The samples were pooled in an equimolar ratio and sent to the Plateforme d’Analyses Génomiques of the Institut de Biologie Intégrative et des Systèmes (Québec, Canada) for sequencing on an Illumina MiSeq platform.

### 2.4. Bioinformatical Analyses

13,075,423 raw reads from the V3−V4 16S region were analyzed through the dada 2 pipeline [32]. Quality control of reads was processed through the filterAndTrim() function by using the following parameters: 270 for the read truncation length, 2 as the phred score threshold for total read removal, and a maximum expected error of 2 for forward reads and 4 for reverse reads. The filtered reads were then fed to the error rate learning, dereplication and amplicon sequence variant (ASV) inference steps using the functions learnErrors(), derepFastq() and dada(). Chimeric sequences were removed using the removeBimeraDenovo() function with the “consensus” method parameter. Taxonomic classification was done through the assignTaxonomy() function using the SILVA132 database as a reference. A total of 2946 ASVs was obtained. Unidentified ASVs and those representing less than 0.0005% of relative abundance were filtered out. At the end of this process, 2423 ASVs were kept for statistical analyses.

### 2.5. Statistical Analysis

Statistical analyses were performed using R software (v 3.3.1, Vienna, Austria). All *p*-values were adjusted with the Benjamini-Hochberg method to reduce the false discovery rate.

Relative abundance plots were generated using only bacterial families having a value of 0.5% or more that were present in at least 25% of the samples. The mean relative abundance of each bacterial family was calculated for each sampling time. Data were fitted using a mixed linear model that included the apiary as a random factor. An ANOVA was performed on the model, followed by Tukey’s HSD test to see if any group differed significantly from the others.

The α-diversity of the honeybee microbiota informs us on the bacterial diversity found in individual samples. It was measured using two indexes, Chao1 and the Shannon index (using the vegan package in R). Comparisons of the indexes between sampling times were carried out as for the relative bacterial abundance.

To determine the β-diversity of the samples, both unweighted and weighted UniFrac distance metrics were used. The UniFrac distance metric uses phylogeny to compare bacterial communities between samples. Unweighted UniFrac distance only considers the presence of bacterial taxa, and weighted UniFrac also accounts for their abundance. A PERMANOVA was carried out to compare the microbial communities between sampling times. A PCoA for each metric was performed in order to visualize the clustering of the microbial samples.

Co-occurrence and co-avoidance of bacterial taxa were calculated with Spearman correlations (using the Hmisc package in R) and *p*-values were corrected with FDR for multiple comparison bias. Positive correlations indicated co-occurrence and negative correlations indicated co-avoidance. Networks were generated with Cytoscape (v.3.7.2, Boston, MA, USA) using correlations with R^2^ values higher or equal to |0.3|.

## 3. Results

### 3.1. Overall Microbiota Composition

**Figure 1:**
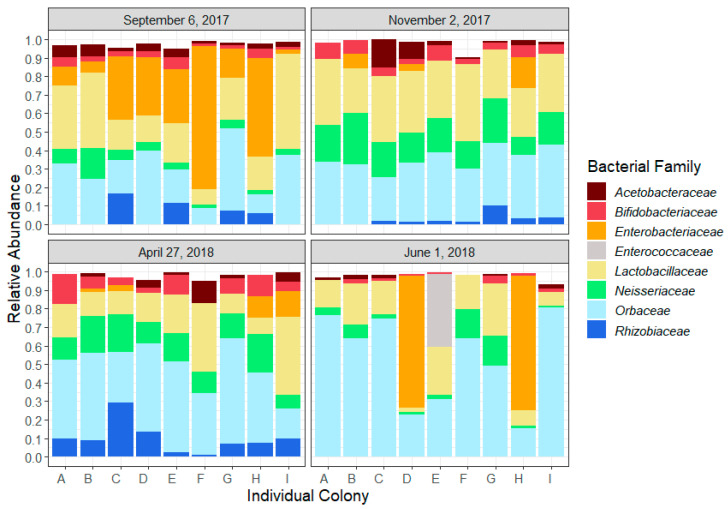
Relative abundance of the core microbiota of the nine colonies (A–I) at the four key sampling moments: September 6, after the last honey flow; November 2, before entering wintering room; April 27, after wintering and removal from wintering room; June 1, during first honey flow of 2018. Bacterial families representing at least 0.5% of the total abundance in 25% of the samples of more are presented.

Bacterial analysis using V3−V4 16S rDNA amplicon libraries revealed that over 90% of the honeybee bacterial microbiota sampled in the control group is dominated by seven bacterial families (Figure 1): *Acetobacteriaceae* (*Bombella* sp., *Commensalibacter* sp.), Bifidobacteriaceae, *Enterobacteriaceae* (*Arsenophonus* sp., *Pantoea* sp.), *Lactobacillaceae*, *Neisseriaceae* (*Snodgrassella* sp.), *Orbaceae* (*Frischella* sp., *Gilliamella* sp.) and *Rhizobiaceae* (*Bartonella* sp.). Together, they constitute the core microbiota of these honeybees. In June, *Enterococcaceae* represented nearly 40% of the gut microbiota of colony E. Since this was the only colony in which this bacterial family was found, we removed it from the analyses.

### 3.2. Seasonal Trends

The honeybee gut microbial composition followed a seasonal trend related to the key sampling dates (Figure 1) and significant differences in relative abundance of bacterial families were observed between sampling times (Table 1). In fall, from September to November, the relative abundance of *Neisseriaceae* increased (t-ratio = −5.345, *p* < 0.0001), while *Enterobacteriaceae* abundance decreased slightly (t-ratio = 2.747, *p* = 0.06). Between November and April, the relative abundance of *Rhizobiaceae* increased (t-ratio = −2.894, *p* = 0.02). In spring, from April to June, we observed a reduction of three core constituents: *Bifidobacteriaceae* (t-ratio = 4.319, *p* = 0.0009), *Neisseriaceae* (t-ratio = 3.683, *p* = 0.0014) and *Rhizobiaceae* (t-ratio = 3.908, *p* = 0.002). From September to June, *Orbaceae* increased significantly (t-ratio = −3.711, *p* = 0.005). The only bacterial family that remained stable for the entire study was *Acetobacteriaceae*.

**Figure 2:**
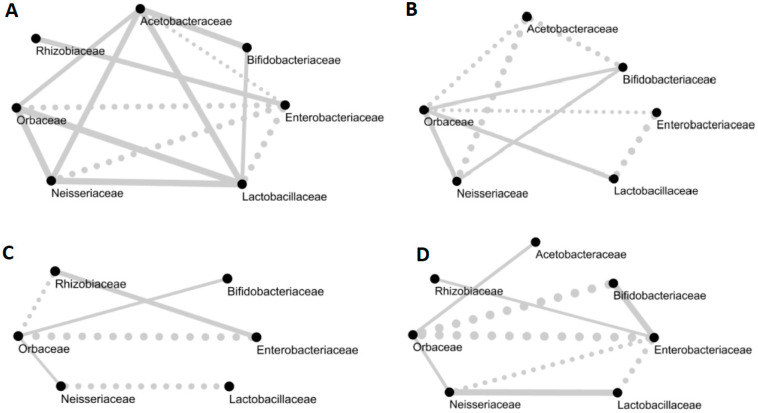
Networks of the core bacterial families of the honeybee gut on (**A**) September 6, (**B**) November 2, (**C**) April 27 and (**D**) June 1. Full lines indicate a positive correlation and dotted lines indicate negative correlations. Correlations under 0.3 are not displayed in the networks. The thickness of the line is proportional to the value of the correlation between two families.

We observed many co-occurrence and co-avoidance trends (Figure 2), but none were significant. Members of *Orbaceae* were positively correlated to *Neisseriaceae* and *Bifidobacteriaceae* in September, November and April, but there was a negative correlation between *Orbaceae* and *Bifidobacteriaceae* in June. The abundance of *Enterobacteriaceae* was negatively correlated with most taxa during our study, except *Rhizobiaceae*. However, in June, *Enterobacteriaceae* was positively correlated to *Bifidobacteriaceae*.

**Figure 3:**
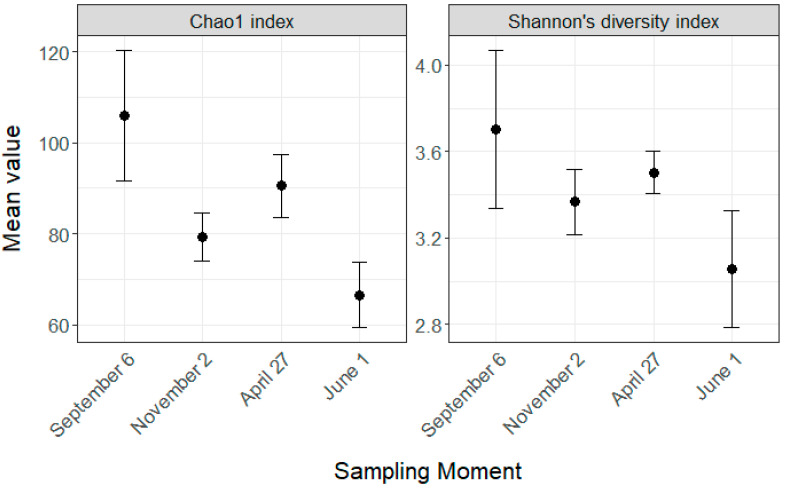
Alpha diversity of the gut microbiota regarding the four key sampling moments. Diversity was measured with Chao1 and Shannon’s diversity index. The overwintering period was from November 22 to April 20. Error bars indicate 95% confidence intervals.

During our study, the sampling time impacted the two indexes we used to assess α-diversity (Figure 3), Chao1 (F = 13.26, *p* < 0.0001) and the Shannon index (F = 4.68, *p* = 0.008). Chao1 was significantly lower in November compared to September (*p* < 0.001), remained stable during the overwintering period (*p* = 0.092) and decreased significantly between April and June (*p* = 0.002). Shannon’s diversity index followed the same trend as Chao1, but only the difference between September and June was significant (*p* = 0.0059).

**Figure 4:**
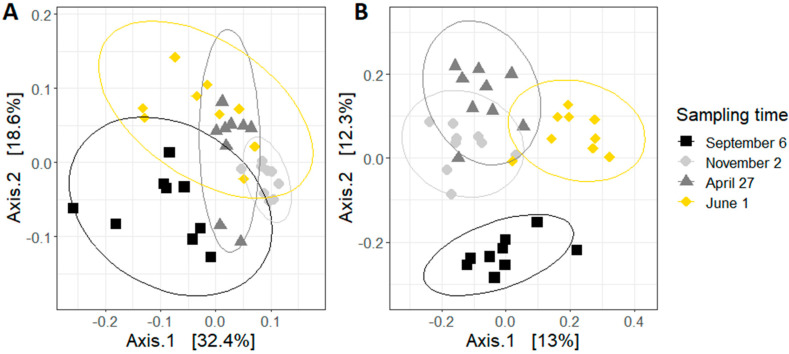
Principal coordinate analysis (PCoA) based on (**A**) unweighted and (**B**) weighted UniFrac distance of samples. Each point represents a colony. Ellipses indicate the 95% confidence intervals.

The principal coordinate analysis (PCoA) plots were generated using an unweighted or weighted UniFrac distance metric (Figure 4). Both plots show that samples are clustered by sampling time. The PCoA plot using unweighted UniFrac distances, which only considers the presence of bacterial species, shows that there is an important difference between the bees sampled in September and the other samples. This difference is smaller in the weighted Unifrac PCoA plot, where the abundance of each bacterial specie is also accounted for. Based on the results of the pairwise PERMANOVA, the sampling time did impact both weighted and unweighted UniFrac distance. All pairwise comparisons between sampling times were significant (*p* < 0.01).

## 4. Discussion

This study aimed to determine the dynamic of the honeybee gut microbiota in a northern temperate climate at four key times from September to June. During this period of the year, honeybees feed on a poor diet mainly consisting of sugar syrup and aged beebread. Moreover, honeybees are confined in their hives, which makes it impossible for them to flush their intestinal content. These two features specific to the wintering period are likely to impact the honeybee gut microbiota. Overall, our results reveal a strong influence of the sampling time on both alpha (Chao1, Shannon) and beta (UniFrac) diversity metrics of the microbiota, as well as its composition.

As expected, the bacterial composition of the microbiota was consistent with the findings of other studies, as the same bacterial families formed the core gut microbiota [33,34]. In addition, the gut microbiota of the colonies monitored in the present study were dominated by *Enterobacteriaceae*, especially in September. Most strains from this family were identified as *Arsenophonus* sp. and *Pantoea* sp. Strains belonging to *Enterobacteriaceae* are often associated with gastrointestinal diseases in mammals [35,36]. In the honeybee, the *Enterobacteriaceae* family is positively correlated with gut dysbiosis [33] and unhealthy colonies [37]. In one study on colonies suffering from Colony Collapse Disorder, the abundance of *Arsenophonus* sp. was abnormally high [38]. While the direct impact of the presence of *Enterobacteriaceae* is still unknown, its abundance in the gut of the honeybees could indicate a health issue that should be investigated further.

We also observed that *Enterobacteriaceae* was negatively correlated with *Lactobacillaceae*, *Orbaceae* and *Neisseriaceae*. Members of these beneficial bacterial families contribute to the innate immune system of the honeybee by stimulating the production of antimicrobial peptides [39]. These molecules could inhibit the growth of *Enterobacteriaceae*, explaining the antagonist relationship observed here.

In our study, the composition of the honeybee gut microbiota followed a seasonal trend. The abundance of *Rhizobiaceae* increased between November and April, but nearly disappeared in spring, after the colonies were taken out of the wintering room. This taxon is frequently found in the honeybee gut, but its impact on the health of the host remains unknown [23]. Interestingly, high levels of *Rhizobiaceae* have previously been associated to bees fed only with sugar syrup [40]. Not surprisingly, our overwintering honeybees, almost exclusively fed with sugar syrup, exhibited a high level of *Rhizobiaceae*. Furthermore, the decrease of *Rhizobiaceae* observed in April coincided with fresh nectar intake. It is therefore very likely that the transition from sugar syrup triggered the important reduction of *Rhizobiaceae* we observed.

Members of *Neisseriaceae*, mostly represented by *S. alvi*, also followed a seasonal trend: an increase in fall, followed by a significant reduction in spring. The bacterium *S. alvi* is known to protect the honeybee gut against opportunistic pathogens by maintaining the intestinal environment anoxic [11] and forming a protective mucopolysaccharide layer on top of the epithelial cells [41]. During the winter season, the bees are clustered within the hive and do not defecate. Therefore, the increase of *Neisseriaceae* during this period could protect their gut against potential pathogens accumulated in the feces until they can excrete them in spring.

We noted that the presence of *Neisseriaceae* was negatively correlated to that of *Enterobacteriaceae*. It has been shown that the availability of oxygen limits the growth of *Enterobacteriaceae* [42]. Here, we hypothesize that the abundance of *Enterobacteriaceae* depended partly on the presence of *S. alvi*, which regulates the amount of oxygen available in the gut. However, in fall, the decrease of *Enterobacteriaceae* could be related to the anti-varroa treatments administered in October (Thymovar^®^) and November (oxalic acid). Members of the *Enterobacteriaceae* family are among the main symbionts of *Varroa destructor* [18,43], and because of horizontal transfer between the mites and the honeybees [44], parasitized bees usually harbor higher levels of *Enterobacteriaceae* strains in their gut. Thus, lowering the infestation levels with varroacides may have reduced the abundance of *Enterobacteriaceae*, allowing the population of *S. alvi* to increase. However, laboratory trials are needed to confirm the antagonistic relationship between these two bacterial taxa.

Between September and November, colonies were treated twice against *V. destructor* with two acaricides, Thymovar^®^ and oxalic acid, which could explain some of the bacterial shifts we observed during this period. In fact, exposure to common acaricides is known to alter the bees’ gut microbiota [45]. Coumaphos triggers an increase of *Bifidobacteriaceae* in the gut of treated bees, while tau-fluvanilate influences both *Rhizobiaceae* and *Enterobacteriaceae* abundance. Moreover, exposure to thymol, the active compound of Thymovar^®^, was observed to alter the abundance of many *Lactobacillaceae* strains in chickens [46].

The microbial richness and diversity of the gut microbiota were lower in June than in September, which is consistent with previous observations [14]. Moreover, the comparison of temporal samples using unweighted and weighted UniFrac metrics showed that the higher diversity measured in September samples was mostly explained by rare taxa. Such a high diversity and quantity of rare taxa observed at the end of the foraging season are both likely due to the bees’ access to an abundance of food harboring a variety of microorganisms that are able to colonize their gut, temporarily or permanently [30]. From November to April (during the wintering period), we observed that the diversity of the microbiota remained stable. This result is not surprising: from November to April, bees are confined in their hives, with no fresh food brought to the colony. Consequently, there are no new microorganisms present in the environment to populate the gut of the bees.

The reduction of the bacterial diversity we observed during our study could be partially explained by the varying growth rate of the colonies. Recently, Ribières et al. compared the gut microbial diversity of non-thriving hives, characterized by slow brood development and low honey production, to that of thriving hives [47]. They found that non-thriving hives had a significantly lower gut diversity that thriving hives. At the beginning of the overwintering period, honeybee colonies reduce their brood rearing and honey production, which is similar to non-thriving colonies. However, in spring, even if colonies increase their brood rearing and honey production, we noted that the bacterial diversity still declined. It is possible that environmental factors specific to the overwintering period had a greater impact on the diversity of the gut microbiota than the growth rate of the colonies, hence the opposite observations.

With this study, we showed that the relative abundance of many bacterial taxa that make up the honeybee microbiota, as well as their diversity, follow a seasonal trend. As discussed, it is likely that the bacterial community was modulated by the weather, nutritional and behavioral changes occurring before and after winter. However, the contribution of each individual factor to the modulation of the honeybee microbiota, and consequently honeybee health, needs to be investigated. Trials using caged honeybees could allow each factor to be isolated, so that their individual and collective impact on bee microbiota, health and physiology can be monitored. The results would allow us to develop innovative bee-specific probiotic formulas.

## Figures and Tables

**Table 1:** Statistical analyses and pairwise comparisons of relative abundance of each bacterial family forming the core microbiota between the four sampling times: September 6, after the last honey flow; November 2, before entering wintering room; April 27, after wintering and removal from wintering room; June 1, during first honey flow of 2018. Sampling times are significantly different if they are identified with different letters.

				Pairwise Comparison			
		F-Value	*p*-Value	Sept. 6	Nov. 2	Apr. 27	Jun. 1
Bacterial family	*Acetobacteriaceae*	1.28	0.29	A	A	A	A
	*Bifidobacteriaceae*	7.15	0.0009	AB	BC	C	A
	*Enterobacteriaceae*	3.21	0.036	A	B	AB	AB
	*Lactobacillaceae*	3.59	0.024	AB	A	AB	B
	*Neisseriaceae*	14.41	<0.0001	A	B	B	A
	*Orbaceae*	5.02	0.006	A	A	AB	B
	*Rhizobiaceae*	5.51	0.003	AB	A	B	A
